# Klotho inhibits growth and promotes apoptosis in human lung cancer cell line A549

**DOI:** 10.1186/1756-9966-29-99

**Published:** 2010-07-19

**Authors:** Bo Chen, Xueli Wang, Weihong Zhao, Jianqing Wu

**Affiliations:** 1 Department of Geriatrics, The First Affiliated Hospital of Nanjing Medical University, Nanjing 210029, Jiangsu, China

## Abstract

**Background:**

Klotho, as a new anti-aging gene, can shed into circulation and act as a multi-functional humoral factor that influences multiple biological processes. Recently, published studies suggest that klotho can also serve as a potential tumor suppressor. The aim of this study is to investigate the effects and possible mechanisms of action of klotho in human lung cancer cell line A549.

**Methods:**

In this study, plasmids encoding klotho or klotho specific shRNAs were constructed to overexpress or knockdown klotho in vitro. A549 cells were respectively treated with pCMV6-MYC-KL or klotho specific shRNAs. The MTT assay was used to evaluate the cytotoxic effects of klotho and flow cytometry was utilized to observe and detect the apoptosis of A549 cells induced by klotho. The activation of IGF-1/insulin signal pathways in A549 cells treated by pCMV6-MYC-KL or shRNAs were evaluated by western blotting. The expression levels of bcl-2 and bax transcripts were evaluated by quantitative reverse transcription-polymerase chain reaction (qRT-PCR).

**Results:**

Overexpression of klotho reduced the proliferation of lung cancer A549 cells, whereas klotho silencing in A549 cells enhanced proliferation. Klotho did not show any effects on HEK-293 cells. Klotho overexpression in A549 cells was associated with reduced IGF-1/insulin-induced phosphorylation of IGF-1R (IGF-1 receptor)/IR (insulin receptor) (*P *< 0.01). Overexpression of klotho can promote the apoptosis of A549 cells (*P *< 0.01). Overexpression of klotho, a bcl family gene bax, was found up-regulated and bcl-2, an anti-apoptosis gene, was found down-regulated (*P *< 0.01). In contrast, bax and bcl-2 were found down-regulated (*P *< 0.05) and up-regulated (*P *< 0.01), respectively when silencing klotho using shRNAs.

**Conclusions:**

Klotho can inhibit proliferation and increase apoptosis of A549 cells, this may be partly due to the inhibition of IGF-1/insulin pathways and involving regulating the expression of the apoptosis-related genes bax/bcl-2. Thus, klotho can serve as a potential tumor suppressor in A549 cells.

## Background

Aging is the greatest risk factor for cancer. About 77% of all cancers are diagnosed in people over 55 years old, with men facing a 50% chance of developing cancer, whereas women having a 35% chance. Thus, with the aging population increasing, it is expected that cancer will become an enormous challenge. Lung cancer is the leading cause of cancer deaths worldwide because of its high incidence and mortality, with 5-year survival rates approximately 10% for non-small cell lung cancer (NSCLC) [1]. It is urgent to investigate the mechanism of tumorigenesis to improve survival rate.

Recently, klotho, a new anti-aging gene, has gained great attention. The klotho gene plays a critical role in regulating aging and the development of age-related diseases in mammals: Loss of klotho can result in multiple aging-like phenotypes [2], while overexpression of klotho gene extends lifespan by 20-30% [3]. The klotho gene is composed of 5 exons [4,5] and encodes a type-I single-pass transmembrane protein (1014-amino acid-long). The intracellular domain is short (10-amino acid-long) and no known functional domains exist. The extracellular domain is composed of two domains, termed KL1 and KL2, with weak homology. Each domain has homology to family 1 glycosidases, including lactose-phlorizin hydrolase of mammals and β-glucosidases of bacteria and plants [2,6]. These enzymes have exoglycosidase activity that hydrolyzes β-glucosidic linkage in saccharides, glycoproteins, and glycolipids. However, recombinant klotho protein did not have β-glucosidase-like enzymatic activity, probably due to critical amino acid residues in putative active centers of klotho protein diverge from those conserved throughout the β-glucosidase family of enzymes [2,6].

Klotho can involve in multiple biological processes, and the precise mechanism was widely and deeply investigated [7]. It is now widely accepted that klotho inhibits insulin and insulin-like growth factor 1 (IGF-1) signaling pathways [3,8]. Moderate inhibition of the insulin/IGF-1 signaling pathways has been viewed as one of the evolutionarily conserved mechanisms for suppressing aging [9]. In addition, klotho functions as a co-receptor for fibroblast growth factor 23 (FGF23), which down-regulates the expression of 1,25-dihydroxyvitamin D3 and phosphate reabsorption [10,11]. Klotho can also increase the resistance to oxidative stress [12]. Furthermore, klotho may protect the cardiovascular system by increasing nitric oxide (NO) production [13].

Multiple lines of evidence suggest the involvement of the IGF-1/insulin pathways across a range of malignancies, including both NSCLC and small cell lung cancer (SCLC) [14-17], and inhibition of IGF-1 signaling pathway is a potential therapy for human lung cancer [18].

Intriguingly, a recently published research suggests that klotho serves as a potential tumor suppressor and identify it as an inhibitor of the IGF-1 pathway and activator of the FGF pathway in human breast cancer [19]. In this study, we detected changes in biological behavior after overexpression or knockdown of klotho in lung cancer cell line A549, and found that it also acts as a potential tumor suppressor in lung cancer.

## Materials and methods

### Constructs

The MYC-tagged klotho expression vector (pCMV6-MYC-KL) and its entry vector (pCMV6) were designed and purchased from OriGene (Rockville, MD, USA). Klotho-directed stealth shRNA duplex oligoribonucleotides and control shRNAc were also designed and purchased from OriGene (Rockville, MD, USA). And their sequences are as follows:

sh-1: CCTAAGCTCTCACTGGATCAATCCTCGAA;

sh-2: CTGAGGCAACTGCTTTCCTGGATTGACCT;

sh-3: GGTCACTCACTACCGCTTCTCCATCTCGT;

sh-4: GTTACAGCATCAGGCGTGGACTCTTCTAT;

shRNAc, scrambled: Non-effective 29-mer scrambled shRNA cassette in pRS plasmid

### Cells culture and transfections

The NSCLC A549 and the noncancerous human embryonic kidney (HEK) 293 cell lines were purchased from ATCC (Manassas, VA, USA), and were maintained in Dulbecco's Modified Eagle's Medium (DMEM) containing 10% fetal bovine serum (FBS), and cultured in a humidified atmosphere of 95% air and 5% CO_2 _at 37°C. All transfections used LipofectAMINE 2000 (Invitrogen, CA, USA). Stable clones were generated by selection in complete culture medium containing 700 μg/ml G418.

### RNA extraction and quantitative real-time RT-PCR

Total RNA was extracted from cells with Trizol reagent (Invitrogen, CA, USA) according to the manufacturer's instructions. Gene expressions were detected by quantitative real-time RT-PCR (qRT-PCR) using the standard SYBR Green RT-PCR kit (Takara, Dalian, China) according to the manufacture's instructions. Briefly, the cDNA was synthesized using the RevertAid First-Strand cDNA Synthesis kit (Fermentas, Vilnius, Lithuania) according to the manufacturer's protocol. The specific primer pairs and the amplified products are as follows: *klotho *(104 bp), sense: 5'-GCTCTCAAAGCCCACATACTG -3'; antisense: 5'-GCAGCATAACGATAGAGGCC -3'; *bcl-2 *(182 bp), sense: 5'-GTGTGGAGAGCGTCAACC -3'; antisense: 5'-CTTCAGAGACAGCCAGGAG -3'; *bax *(91 bp), sense: 5'-ATGCGTCCACCAAGAAGC- 3'; antisense: 5'-ACGGCGGCAATCATCCTC -3'; *β-actin *(205 bp) as an internal, primers, sense: 5'-TGACGTGGACATCCGCAAAG -3'; antisense: 5'-CTGGAAGGTGGACAGCGAGG -3'. The relative levels of gene mRNA transcripts were normalized to the control *β-actin*. Relative gene expression was quantified using the GraphPad Prism 4.0 software (GraphPad Software, San Diego, CA, USA). PCR consisted of initial denaturation at 94°C for 5 min, followed by 30 reaction cycles (30 seconds at 94°C, 30 seconds at 60°C, and 30 seconds at 72°C) and a final cycle at 72°C for 10 min.

### Western blot analysis

The cells were lysed in 0.1 ml lysis buffer (0.1% SDS, 1% NP-40, 50 mM HEPES, pH 7.4, 2 mM EDTA, 100 mM NaCl, 5 mM sodium orthovanadate, and 1% protease inhibitor mixture set I; Calbiochem) on ice for 30 min, and lysates were cleared by centrifugation at 12,000 rpm for 15 min. Proteins were separated in 10% SDS-PAGE and electroblotted onto polyvinylidene difluoride membrane, blocked for 1.5 hr at room temperature in 5% non-fat milk or 1% BSA, and probed with anti-IGF-1β receptor (111A9) and phospho-IGF-1R (Y1135/1136), phospho-IR (Y1150/1151), and anti-β-actin (Cell Signaling Technology, MA, USA) antibody. Following incubation with the corresponding peroxidase-conjugated secondary antibodies, Chemiluminent detection was performed with the ECL kit (Pierce, Rockford, IL, USA).

### MTT viability assay

Cell proliferation was evaluated by a modified 3-(4,5-dimethylthiazol-2-yl)-2,5-diphenyltetrazolium bromide (MTT) assay. The test cells in exponential growth were plated at a final concentration of 8 × 10^3 ^cells/well in 96-well culture plates for different culture time. MTT (20 μl, 10 mg/ml) was then added. After an additional 4 hr of incubation, the reaction was terminated by removal of the supernatant and addition of 150 μl DMSO for 10 min. Optical density (OD) of each well was measured at 570 nm using ELISA reader (ELx808 Bio-Tek Instruments, Winooski, USA).

### Detection of apoptosis by flow cytometry

Cells were stained with fluorescein isothiocyanate (FITC) labeled annexin-V, and simultaneously with propidium iodide (PI) stain, to discriminate intact cells (annexin-/PI-) from apoptotic cells (annexin+/PI-), and necrotic cells (annexin+/PI+). A total of 1.0 × 10^6 ^cells were washed twice with ice-cold PBS and incubated for 30 min in a binding buffer (1 μg/ml PI and 1 μg/ml FITC labeled annexin-V), respectively. FACS analysis for annexin-V and PI staining was performed by flow cytometer (Coulter, Beckman, CA, USA). All experiments were performed in triplicate.

### Statistical analysis

Data were expressed as mean ± SD. Statistical analysis was performed using SPSS software (Release 13.0, SPSS Inc.). The difference between two groups was analyzed by the Student's *t*-test. A value of *p *< 0.05 was considered as statistical significance.

## Results

### Klotho expression after transfection with pCMV6-MYC-KL or shRNA

**Figure 1:**
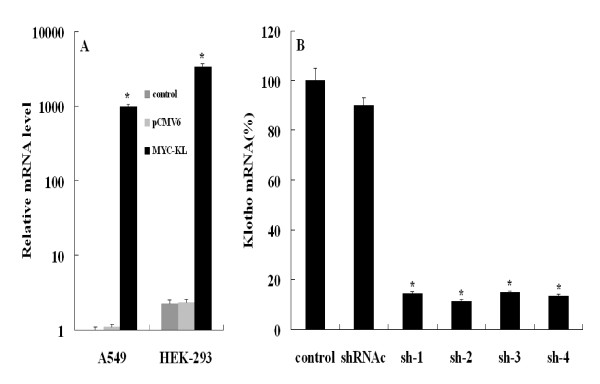
**Relative klotho gene transcripts by qRT-PCR**. (A) A549 and HEK-293 cells transfected with either MYC-tagged klotho expressison vector (MYC-KL) or an entry vector (pCMV6). (B) A549 cells transfected with four klotho directed-shRNAs and a negative control-shRNA (shRNAc). Data shown are the mean results ± SD of a representative experiment performed in triplicate (*n *= 3), *indicates *p *< 0.01. Statistical comparisons showed that our klotho expression plasmid and klotho-specific shRNA could work efficiently.

To determine the effects of overexpression or knockdown of klotho in A549 and HEK-293 cells, we generated a MYC-tagged klotho expressison vector (pCMV6-MYC-KL), four klotho directed-shRNAs and a negative control-shRNA (shRNAc). After the cells were transfected with pCMV6-MYC-KL or klotho specific-shRNAs, the mRNA levels of klotho were analyzed with qRT-PCR. Obviously, the levels of klotho mRNA transcripts were highly elevated in pCMV6-MYC-KL-transfected cells when compared with pCMV6 **(**Figure 1A, whereas in klotho direced-shRNA cells significantly decreased by ~ 89% compared with shRNAc (*P *< 0.01). The results indicate that all four shRNAs are working well, and the effects of sh-2 and sh-4 are very similar and more robust than the other two shRNAs (Figure 1B). Thus, our klotho expression plasmid and klotho-specific shRNAs worked efficiently.

### Klotho inhibits lung cancer cell growth and may involve in IGF-1-induced A549 proliferation

**Figure 2:**
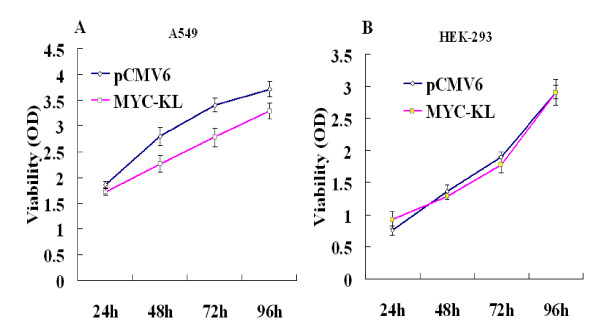
**Effects of klotho on A549 and HEK-293 cells growth dynamics determined by MTT**. (A) and (B) are A549 and HEK-293 cells transfected either with pCMV6 or with MYC-KL, respectively.

A549 and HEK-293 cells were transfected with either pCMV6-MYC-KL vector or empty vector (pCMV6). To assess the effects of klotho expression, A549 clones, which expressed either pCMV6 or pCMV6-MYC-KL, were generated. The proliferation of klotho-expressing cells, as evaluated by MTT assay, was significantly inhibited when compared with the controls. The inhibition rates ranged from 7% to 20%, and the results are shown in Figure 2A (*P *< 0.05). However, we did not find any significance in HEK-293 cells after overexpression of klotho (*P *> 0.05; Figure 2B).

**Figure 3:**
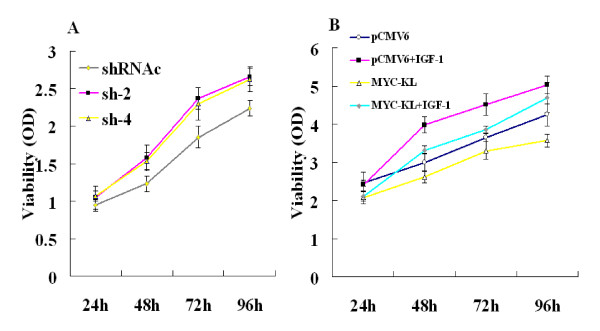
**Effects of klotho on A549 cells growth dynamics determined by MTT**. (A) A549 cells transfected by negative control-shRNA (shRNAc) or klotho-directed shRNAs sh-2 and sh-4. (B) A549 cells were transfected with either MYC-KL or pCMV6, starved for 24 hr and treated by IGF-1 (25 nM) for 24-96 hr.

As we found some klotho expression in A549 cells, we examined the effects of downregulation of klotho in these cells. Four klotho-specific shRNAs were designed and tested for their ability to silence klotho expression in A549 cells, compared with negative control group shRNAc. We investigated the growth condition after transfection with the sh-2 and sh-4, respectively. Following downregulation of klotho, proliferation of A549 cells, as assessed by MTT assay, elevated by 11% to 28% and 13% to 25% using sh-2 and sh-4, compared with shRNAc, respectively (Figure 3A).

It is widely accepted that klotho inhibits the activation of the IGF-1 pathway [3,8], which has an important function in lung cancer tumorigenesis, and IGF-1 induces A549 cells proliferation [20]. To test the ability of klotho to modulate IGF-1-induced proliferation and survival, A549 cells were transiently transfected with either pCMV6 or pCMV6-MYC-KL and grown in 0.5% serum with either IGF-1 or a control vehicle for 24-96 hr. Klotho transfection obviously inhibited cell proliferation in the untreated cells, and this inhibition was only mildly restored following addition of IGF-1 to the cells. Thus, whereas IGF-1 increased cell proliferation by up to 33% in control pCMV6-transfected cells, cell proliferation in the pCMV6-MYC-KL-transfected cells increased by only 11% (Figure 3B).

### Klotho inhibits the activation of the IGF-1/insulin pathways and is directly associated with IGF-1R in lung cancer cells

**Figure 4:**
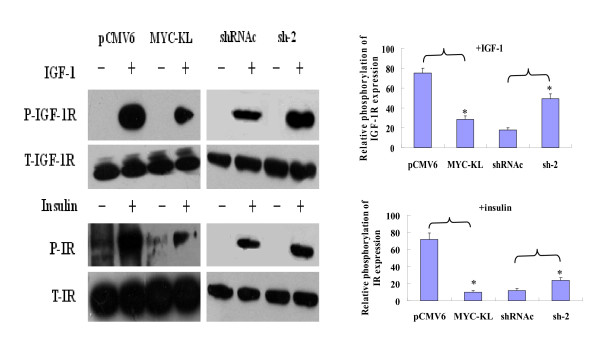
**Downregulation of the IGF-1/insulin pathways by klotho in lung cancer cell line A549**. A549 cells were transfected with either MYC-KL or control vector pCMV6. After 24 hr, cells were serum-starved for 24 hr and treated with IGF-1 (10 min, 25 nM) or insulin (10 min, 100 nM). Following treatment, cells were harvested and proteins were resolved and immunoblotted using antibodies either directed against phospho (P) and total (T) IGF-1R or phospho (P) and total (T) insulin-R (IR). Similar treatment was done when silenced the klotho of the cells using sh-2 or control shRNAc. Data shown are the mean results ± SD of a representative experiment performed in triplicate (*n *= 3), *indicates *p *< 0.01.

We studied the effect of klotho on IGF-1 pathway activation in A549 lung cancer cells, which express high levels of IGF-1R and show an enhanced proliferation following IGF-1 treatment. A549 cells were transfected with either pCMV6-MYC-KL or pCMV6, starved for 24 hr, treated with IGF-1 (10 min, 25 nM) and analysed using western blotting for the expression and phosphorylation of IGF-1R. Klotho overexpression in A549 cells was associated with reduced phosphorylation of IGF-1R (*P *< 0.01). The effects of overexpression of klotho on the insulin (10 min, 100 nM) pathway were also examined, and similar to IGF-1 activation, klotho overexpression in A549 cells was associated with reduced phosphorylation of insulin receptor (IR, *P *< 0.01), indicating that klotho also inhibited the activation of the insulin pathway in A549 cells. We further studied the effect of klotho knockdown in A549 cells using sh-2, and found a significant increase in IGF-1R/IR phosphorylation following IGF-1/insulin stimulation in sh-2-transfected cells compared with siRNAc-transfected cells. The results were shown in Figure 4.

### Klotho-induced apoptosis of A549 cells

**Figure 5:**
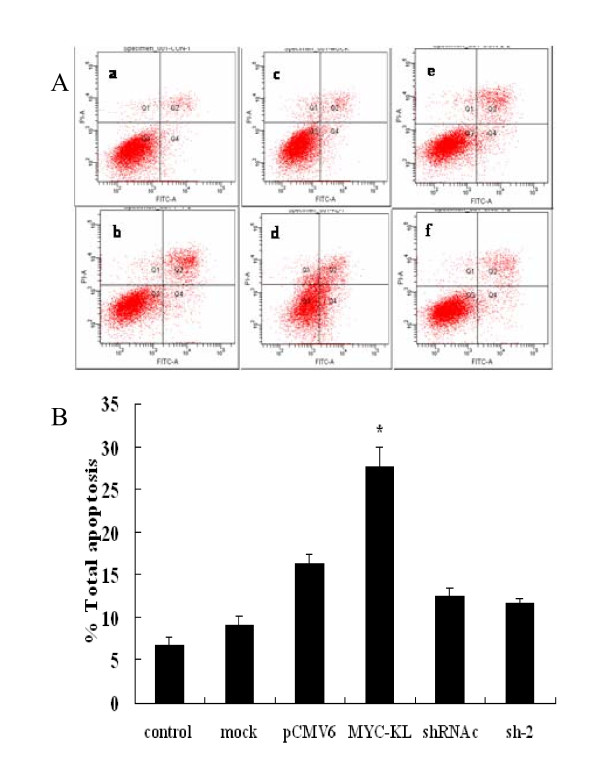
**Induction of A549 cells apoptosis after overexpression of klotho**. (A) Figures of apoptosis by flow cytometry. a, b, c, d, e and f indicate control, mock, pCMV6, MYC-KL, shRNAc and sh-2 groups, respectively. (B) The data present the average number of apoptotic cells (± SD) in three independent experiments. pCMV6 *vs *MYC-KL, * indicates *p *< 0.01.

To determine the effects of overexpression or downregulation of klotho on the klotho-induced apoptosis in A549 cells, the rate of apoptosis was evaluated by flow cytometry analysis. As shown in Figure 5, the effects of klotho-induced apoptosis were investigated in pCMV6 cells as well as cells transfected with pCMV6-MYC-KL, sh-2 or shRNAc. We found that the significantly elevated percentage of apoptosis in the cells transfected with klotho compared with the control cells (*P *< 0.01), while there was no significant difference between sh-2-transfected and shRNAc-transfected cells (*P *> 0.05).

### Apoptosis-related gene expression in the klotho-induced apoptosis

**Figure 6:**
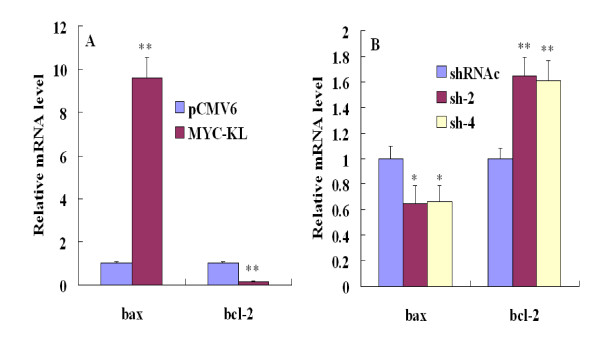
**Influence of down-stream genes expression in klotho-induced apoptosis**. (A) After tansfected with MYC-KL, bax and bcl-2 genes transcripts were found up-regulated and down-regulated, respectively. (B) Compared with shRNAc group, bax and bcl-2 genes transcripts were found down-regulated and up-regulated respectively in sh-2/4-transfected group. Data shown are the mean results ± SD of a representative experiment performed in triplicate (n = 3), *indicates *p *< 0.05; **indicates *p *< 0.01.

We next investigated potential pathways involved in klotho-induced apoptosis. As shown in Figure 6, overexpression of klotho, a bcl family gene bax, was found up-regulated compared with pCMV6-transfected cells while down-regulated when transfected with klotho specific-shRNA sh-2 compared with shRNAc-transfected cells. In contrast, bcl-2, an anti-apoptosis gene, was found down-regulated when overexpression of klotho, while up-regulated when downregulation of klotho using sh-2. Similar results were obtained when comparing sh-4 group with shRNAc group. These results showed that bax and bcl-2-related apoptosis pathways may involve in the klotho-induced apoptosis.

## Discussion

Recent studies demonstrated that mutation of a single gene in chromosome 13, which is now widely identified as klotho, causes extensive aging phenotypes including arteriosclerosis, vascular calcifications, soft tissue calcifications, emphysema, hypoactivity, gonadal dysplasia, infertility, skin atrophy, ataxia, hypoglycemia and severe hyperphosphatemia. It may be associated with increased concentrations of 1,25(OH)2D3, an essential vitamin for calcium metabolism [2]. Thus, klotho is widely recognized as an anti-aging gene. In addition to its role in aging, recent research found that it can involve in multiple cell signal pathways with complex roles. In addition to regulating insulin and IGF-1, acting as a co-receptor for FGF23 and resisting to oxidative stress, it also influences several intracellular signaling pathways which underlie the molecular mechanism of klotho function, such as p53/p21 [21], cAMP [22], PKC and Wnt [10] signaling pathways.

Ample clinical and laboratory data indicate a critical role for insulin/IGF-1 signaling in lung cancer. Notably, intracellular presence of insulin is associated with adverse prognosis in human lung adenocarcinoma [17]; High circulating IGF-1 levels are associated with increased risk of lung cancer [16], and inhibition of insulin/IGF-1 signaling inhibits growth of lung cancer cells [20] . Since secreted klotho protein can inhibit the activation of insulin/IGF-1 receptors, we presumed that klotho may also function as a suppressor of lung cancer. In this study, we investigated the effects of klotho in lung cancer cells. We found that the expression of klotho in lung cancer cell line A549 is low, and klotho overexpression inhibits, whereas klotho downregulation enhances, lung cancer cell growth. In addition, we found that overexpression of klotho was associated with reduced phosphorylation of IGF-1R using IGF-1 stimulation, and similar results were found in the evaluation of insulin pathway. Our results consistent with recently published paper which demonstrated that klotho can act as a tumor suppressor and a modulator of the IGF-1 and FGF pathways in human breast cancer [19]. The possible reason may be that IGF-1 pathway involves in tumorigenesis of this two cancer types. In sum, our results indicate that klotho inhibit A549 cells growth partly due to inhibition of IGF-1/insulin pathways.

The regulation of apoptosis is a complex process and involves a number of gene products including bcl-2 protein family and cell cycle-regulatory proteins. The bcl-2 family of proteins, as important regulators in both the inhibition and the promotion of apoptosis, forms ion channels in biological membranes, and this ion channel regulates apoptosis by influencing the permeability of the intracellular membrane of mitochondria [23,24]. It was proposed that the ratio between bcl-2 and bax is more important in the regulation of apoptosis than the level of each bcl-2 family protein alone [25]. Our data indicated that treatment with klotho markedly decreased the mRNA levels of bcl-2 and increased bax expression, while the opposite results were obtained when silencing klotho. Thus, the bax/bcl-2 ratio increased with the treatment of klotho. Intriguingly, though the apoptosis-related genes transcripts were all statistically significant between experimental groups and their controls, our flow cytometry results did not show any significance between klotho-specific shRNA groups and shRNAc groups. The possible reason may be gene transcripts are more sensitive and more easily to be detected than the changes in protein and function levels. The apoptosis of A549 cells with low klotho expression may be too weak to observe after knockdown of klotho. In contrast, after forced expression of klotho, the expression of klotho increased several thousand times. Thus, klotho can show effects more obviously, and the apoptosis of A549 cells were more easily to be detected. Moreover, besides bax/bcl-2 signals, there are other mechanisms may take part in klotho-induced A549 cells apoptosis. In addition, forced expression of any protein can cause endoplasmic reticulum (ER) stress, which potentially results in slow proliferation, cell cycle arrest, and/or apoptosis [26]. But, our klotho silencing results may eliminate this possibility. Though having no statistically significant change, the apoptosis of A549 cells tend to decrease after knockdown of klotho. And the changes of apoptosis-related genes bax/bcl-2 also supported that klotho may promote apoptosis of A549 cells. All these results suggested that the expression levels of anti-apoptotic bcl-2 decreased and pro-apoptotic bax increased, which might play a key role in klotho-induced apoptosis in the A549 cells.

## Conclusions

In summary, klotho, a potential tumor suppressor, can inhibit the growth of lung cancer cells A549 and promote their apoptosis, this may be partly due to the inhibition of IGF-1/insulin pathways and involving regulating the expression of the apoptosis-related genes bax/bcl-2. The function of klotho is very complex, and the signal pathways in cancer development are interwound and cross-linking, so the exact role and working mechanisms of klotho in vitro and in vivo are still waiting to be explored. Further study of the biological functions of klotho may be helpful in developing new strategies in lung cancer treatment.

## Competing interests

The authors declare that they have no competing interests.

## Authors' contributions

In our study, all authors are in agreement with the content of the manuscript. Each author's contribution to the paper: BC: First author, Participated in research design, the writing of the paper, the performance of the research and data analysis. JQW: Corresponding author, research instruction, data analysis, development of final manuscript. XLW: the performance of the research and data analysis. WHZ: research instruction, development of final manuscript.
